# Safety and Accuracy of Matrix-Assisted Laser Desorption Ionization–Time of Flight Mass Spectrometry for Identification of Highly Pathogenic Organisms

**DOI:** 10.1128/JCM.01023-17

**Published:** 2017-11-27

**Authors:** James T. Rudrik, Marty K. Soehnlen, Michael J. Perry, Maureen M. Sullivan, Wanda Reiter-Kintz, Philip A. Lee, Denise Pettit, Anthony Tran, Erin Swaney

**Affiliations:** a Bureau of Laboratories, Michigan Department of Health and Human Services, Lansing, Michigan, USA; b Biodefense Laboratory, Wadsworth Center, New York State Department of Health, Albany, New York, USA; c Public Health Laboratory, Minnesota Department of Health, St. Paul, Minnesota, USA; d State Hygienic Laboratory at the University of Iowa, Coralville, Iowa, USA; e Bureau of Public Health Laboratories, Florida Department of Health, Jacksonville, Florida, USA; f North Carolina State Laboratory of Public Health, North Carolina Department of Health and Human Services, Raleigh, North Carolina, USA; g Bureau of the Public Health Laboratory, New York City Department of Health and Mental Hygiene, New York City, New York, USA; h Texas Department of State Health Services Laboratory, Austin, Texas, USA; Cleveland Clinic

**Keywords:** MALDI-TOF, biothreat agents, clinical laboratory, pathogenic organisms, public health

## Abstract

Matrix-assisted laser desorption ionization–time of flight mass spectrometry (MALDI-TOF MS) sample preparation methods, including the direct, on-plate formic acid, and ethanol/formic acid tube extraction methods, were evaluated for their ability to render highly pathogenic organisms nonviable and safe for handling in a biosafety level 2 laboratory. Of these, the tube extraction procedure was the most successful, with none of the tested strains surviving this sample preparation method. Tube extracts from several agents of bioterrorism and their near neighbors were analyzed in an eight-laboratory study to examine the utility of the Bruker Biotyper and Vitek MS MALDI-TOF MS systems and their *in vitro* diagnostic (IVD), research-use-only, and Security-Relevant databases, as applicable, to accurately identify these agents. Forty-six distinct strains of Bacillus anthracis, Yersinia pestis, Francisella tularensis, Burkholderia mallei, Burkholderia pseudomallei, Clostridium botulinum, Brucella melitensis, Brucella abortus, Brucella suis, and Brucella canis were extracted and distributed to participating laboratories for analysis. A total of 35 near-neighbor isolates were also analyzed.

## INTRODUCTION

Matrix-assisted laser desorption ionization–time of flight mass spectrometry (MALDI-TOF MS) is a rapid, sensitive, and cost-effective method that offers an alternative to traditional phenotypic methods for organism identification in clinical laboratories. As this technology becomes more widely used, laboratories must adapt their workflow and validate the technology for routine use.

Recent breaks in biosafety protocol at the Centers for Disease Control and Prevention (CDC) (1, 2), the shipment of inadequately inactivated Bacillus anthracis spores from the Dugway Proving Grounds (3), and the difficulty clinical laboratories experienced when preparing for a potential Ebola event have led to national initiatives to improve laboratory biosafety. The use of risk assessments plays a critical role in this improvement. In this multilaboratory study, we sought to evaluate the ability of three MALDI-TOF MS sample preparation techniques to render several potential agents of bioterrorism (BT) nonviable prior to removal of the organisms from a biosafety cabinet. Previous sample preparation studies (4–7) have produced conflicting results with respect to their ability to adequately inactivate pathogens and may not have utilized manufacturer-recommended methods.

Indeed, validation and/or verification of MALDI-TOF MS software libraries poses another significant dilemma for clinical laboratories (8). The Clinical and Laboratory Standards Institute (CLSI) (9) has recently published some example end-user verification protocols, including a suggested list of organisms for testing, but the protocols still suggest that the final selection of organisms for verification should be compiled by the individual laboratory. This issue may be complicated by the choice of libraries (e.g., Food and Drug Administration [FDA] approved versus research use only [RUO]) that a laboratory elects to use. Most laboratories lack the resources and culture collection to verify every database entry but instead must verify the ability of their system to identify the clinical agents that they most commonly encounter. Due to Select Agent Program regulations, most clinical laboratories do not have access to BT agents and are unable to verify software performance for these agents. Therefore, this study was set up to evaluate the performance of the RUO and FDA-approved software packages offered by Bruker Daltonics (Billerica, MA) and bioMérieux (Durham, NC) using specimens prepared by the tube extraction method and tested in triplicate by eight participating laboratories. In addition, the Security-Relevant (SR) library available on the Bruker instrument was also tested.

## RESULTS

### Safety study.

Overall results of the study are shown in Table 1. Eighty-nine percent of samples contained viable organisms after 1 μl of drying of an organism suspension on a sterile coverslip (“Spot” samples). This suggests that whereas drying alone affects the viability of some organisms, it is insufficient to render samples nonviable in the time frame associated with routine sample preparation. Exposure to air for an extended period may also have contributed to the decreased viability of the Clostridium spp. The reagents used in sample preparation (Spot + Matrix) for the direct and extended direct methods appeared to have little inhibitory effect, with 68% and 71% of the samples remaining viable, respectively. Only 11% of the samples that had been exposed to the tube extraction reagents contained viable organisms. Viable organisms were present on the target for 18% of the samples prepared using the direct and extended direct methods. No viable organisms were found following the tube extraction.

**TABLE 1:** Viability of BT agents following MALDI-TOF sample preparation

Organism(s)	No. of tubes with growth using indicated sample preparation method/no. tested								
	Direct colony			On-plate formic acid			Tube extraction		
	Target	Spot + Matrix	Spot	Target	Spot + Matrix	Spot	Target	Spot + Matrix	Spot
Bacillus anthracis	3/5	5/5	5/5	1/5	5/5	5/5	0/5	1/5	5/5
Burkholderia thailandensis	0/5	5/5	5/5	0/5	5/5	5/5	0/5	0/5	5/5
Clostridium botulinum/Clostridium perfringens	1/5	1/5	3/5	1/5	0/5	2/5	0/5	1/5	4/5
Francisella tularensis	1/5	2/5	4/5	1/5	2/5	5/5	0/5	1/5	5/5
Yersinia pestis	0/4	3/4	4/4	1/4	4/4	4/4	0/4	0/4	3/4
Brucella abortus	0/4	3/4	4/4	1/4	4/4	4/4	0/4	0/4	3/4
Total	5/28	19/28	25/28	5/28	20/28	25/28	0/28	3/28	25/28

### Accuracy study.

Two experiments were performed to eliminate storage and pooling of extracts as potential sources of error. Isolates of Staphylococcus aureus ATCC 29213, Pseudomonas aeruginosa ATCC 27853, and Clostridium perfringens ATCC 13124 were extracted and tested in triplicate. The remaining extract was divided, stored at −20°C, and retested after 30 and 45 days in storage. The identification scores compared across time showed coefficients of variation of 3.9% for S. aureus, 2.8% for P. aeruginosa, and 1.3% for C. perfringens, indicating little deterioration of the extracts during storage. To demonstrate that pooling of extracts did not alter results, isolates of Streptococcus pneumoniae ATCC 49619, Burkholderia cepacia ATCC 17765, and Moraxella catarrhalis C11-11811 were extracted and tested in triplicate and then the remaining extracts for each organism were pooled and retested in triplicate. The coefficients of variation were 0.96% for M. catarrhalis, 1.8% for B. cepacia, and 2.9% for S. pneumoniae for the nonpooled extracts and were 0.61%, 2.1%, and 3.2% for the pooled extracts, respectively.

Since the only manufacturer-approved specimen preparation technique for Vitek MS system is the direct method, 50 random isolates submitted to the laboratory for identification were tested by the direct and tube extraction methods. Results showed that the two extraction methods yielded the same identification 96% of the time, with each sample preparation method providing one incorrect identification.

Identification accuracy results for the BT agents are shown in Table 2 and Table 3 for the Bruker and Vitek platforms, respectively. Results for near neighbors are shown in Table 4 and Table 5. Some participants failed to test the extracts a single time and then reanalyze the spectra using the other libraries; in some instances, the laboratories prepared new targets for each software library. Any result reported as representing no peaks or inadequate spectra was eliminated from data analysis.

**TABLE 2:** Identification of BT agents by Bruker Biotyper^*a*^

Organism (no. of strains tested)	Reported identification	IVD library		RUO library		SR library	
		Mean score (no. of replicates)	Species-level ID% (≥2.0) (no. of replicates)	Mean score (no. of replicates)	Species-level ID % (≥2.0) (no. of replicates)	Mean score (no. of replicates)	Species-level ID% (≥2.0) (no. of replicates)
Bacillus anthracis (6)	No reliable ID	1.13 (90)		1.38 (45)		1.1 (40)	
	Bacillus cereus			1.88 (62)	8.3 (9)		
	Bacillus anthracis					2.08 (68)	49.1 (53)
	Bacillus pseudomycoides			1.56 (1)			
Francisella pestis (6)	No reliable ID	1.47 (8)		1.5 (10)		1.46 (14)	
	Yersinia pseudotuberculosis	2.15 (72)	73.8 (59)	2.2 (98)	81.5 (88)		
	Yersinia pestis					2.16 (94)	82.4 (89)
Francisella tularensis (5)	No reliable ID	1.11 (60)		1.33 (87)		1.34 (46)	
	Francisella tularensis					1.77 (41)	4.6 (4)
Burkholderia mallei (6)	No reliable ID	1.27 (75)		1.56 (47)			
	Burkholderia thailandensis			1.87 (58)	9.3 (10)		
	Burkholderia pseudomallei					2.16 (5)	3.7 (4)
	Burkholderia mallei					2.17 (103)	87 (94)
	Burkholderia vietnamiensis			1.33 (3)			
Burkholderia pseudomallei (6)	No reliable ID	1.27 (74)		1.54 (28)			
	Burkholderia thailandensis			1.84 (79)	5.6 (6)		
	Burkholderia pseudomallei					2.1 (100)	78.5 (84)
	Burkholderia mallei					2.06 (7)	6.5 (7)
Clostridium botulinum (4)	No reliable ID	1.08 (51)		1.47 (26)		1.29 (51)	
	Clostridium sporogenes			1.86 (46)			
	Clostridium botulinum					1.84 (21)	15.3 (11)
Brucella melitensis (2)	No reliable ID	1.04 (18)		1.3 (36)		1.6 (2)	
	Brucella melitensis					2.17 (34)	83.3 (30)
Brucella abortus (2)	No reliable ID	1.03 (23)		1.31 (35)			
	Brucella melitensis					2.17 (35)	82.9 (29)
Brucella suis (1)	No reliable ID	1.04 (12)		1.27 (18)		0 (1)	
	Brucella melitensis					2.17 (17)	94.4 (17)
Brucella canis (1)	No reliable ID	0.75 (12)		1.21 (18)		1.63 (3)	
	Brucella melitensis					2.05 (15)	55.6 (10)

a ID%, percent identity.

**TABLE 3:** Identification of BT agents by Vitek MS

Organism (no. of strains tested)	Reported identification	IVD library		RUO library	
		Mean score(s) (no. of replicates)	Species-level ID% (≥60%) (no. of replicates)	Mean score (no. of replicates)	Species-level ID% (≥60%) (no. of replicates)
Bacillus anthracis (6)	No identification	0 (12)		0 (19)	
	Bacillus thuringiensis/Bacillus cereus/Bacillus mycoides	33.3/33.3/33.3 (36)			
	Bacillus cereus group			94.9 (35)	
Yersinia pestis (6)	No identification	0 (2)		0 (23)	
	Yersinia pseudotuberculosis/Yersinia frederiksenii	51.7/48.2 (7)			
	Yersinia ruckeri/Yersinia pseudotuberculosis/Yersinia frederiksenii	33.3/33.4/33.2 (1)			
	Yersinia pseudotuberculosis	99.9 (17)	58.6 (17)	87.8 (18)	33.3 (18)
	Yersinia pestis	99.9 (1)	3.4 (1)		
	Yersinia spp.			88.8 (13)	
Francisella tularensis (5)	No identification	0 (33)		0 (31)	
	Francisella tularensis			77.5 (14)	24.4 (11)
	Enterobacter cloacae/Enterobacter asburiae	50/50 (1)			
	Streptococcus constellatus	68.3 (1)	2.2 (1)		
	Streptococcus pluranimalium	64.5 (3)	4.4 (2)		
	Vibrio mimicus	33.6 (1)			
	Kocuria varians/Gemella bergeri	33.3/33.4 (1)			
	Cellulosimicrobium cellulans/Streptococcus equi subsp. *equi*	46.2/53.7 (1)			
	Acinetobacter johnsonii	79.8 (2)	4.4 (2)		
	Kytococcus sedentarius	96.1 (1)	2.2 (2)		
Burkholderia mallei (6)	No identification	0 (51)		0 (31)	
	Burkholderia multivorans	99.4 (1)	1.9 (1)		
	Burkholderia spp.			77.8 (6)	
	Streptococcus porcinus	95.3 (2)	3.7 (2)		
	Staphylococcus carnosus			75.2 (1)	1.9 (1)
	Escherichia coli			79.3 (1)	1.9 (1)
	Yersinia spp.			77.8 (1)	
Burkholderia pseudomallei (6)	No identification	0 (54)		0 (47)	
	Burkholderia spp.			76.2 (2)	
	Staphylococcus aureus			77.3 (1)	1.9 (1)
	Streptococcus oralis			86 (1)	1.9 (1)
	Escherichia coli			86.5 (1)	1.9 (1)
	Yersinia spp.			75 (1)	
Clostridium botulinum (4)	No identification	0 (9)		0 (35)	
	Clostridium sporogenes	99.5 (24)	66.7 (24)		
	Mycobacterium bovis/Mycobacterium tuberculosis/Clostridium sporogenes	33/33/33 (3)			
	Candida krusei			79 (1)	2.8 (1)
Brucella melitensis (2)	No identification	0 (16)		0 (7)	
	Brucella spp.			90.2 (11)	
	Enterococcus avium	35.6 (1)			
	Prevotella disiens	99.9 (1)	5.6 (1)		
Brucella abortus (2)	No identification	0 (15)		0 (9)	
	Brucella spp.			83.5 (9)	
	Listeria seeligeri	50.1 (1)			
	Alloiococcus otitis	51.4 (1)			
	Actinomyces radingae/Listeria seeligeri	56.4/43.5 (1)			
Brucella suis (1)	No identification	0 (6)			
	Brucella spp.			89.9 (6)	
Brucella canis (1)	No identification	0 (8)		0 (5)	
	Brucella spp.			89.2 (3)	
	Gordonia rubripertincta/Ralstonia mannitolilytica/Listeria seeligeri	21.4/21.4/21.4 (1)			
	Candida glabrata			90.2 (1)	11.1 (1)

**TABLE 4:** Identification of near neighbors by Bruker Biotyper

Organism (no. of strains tested)	Reported identification	IVD library		RUO library		SR library	
		Mean score (no. of replicates)	Species-level ID% (≥2.0) (no. of replicates)	Mean score (no. of replicates)	Species-level ID % (≥2.0) (no. of replicates)	Mean score (no. of replicates)	Species-level ID % (≥2.0) (no. of replicates)
Bacillus thuringiensis (1)	No reliable ID	1.1 (12)		1.33 (9)		1.12 (9)	
	Bacillus cereus			2.01 (9)	22.2 (4)		
	Bacillus anthracis					2.06 (9)	38.9 (7)
Bacillus circulans (1)	No reliable ID	1.06 (12)		1.33 (5)		1.01 (18)	
	Bacillus circulans			1.87 (13)	16.7 (3)		
Bacillus cereus (1)	No reliable ID	1.15 (12)		1.35 (9)		1.23 (9)	
	Bacillus cereus			2.08 (9)	44.4 (8)		
	Bacillus anthracis					2.14 (9)	50 (9)
Bacillus mycoides (1)	No reliable ID	1.15 (15)		1.61 (7)			
	Bacillus mycoides			1.77 (7)			
	Bacillus anthracis					1.77 (18)	
	Bacillus weihenstephanensis			1.19 (4)			
Bacillus megaterium (1)	No reliable ID	1.12 (12)		1.35 (3)		1.06 (18)	
	Bacillus megaterium			1.98 (15)	55.6 (10)		
Bacillus subtilis (1)	No reliable ID	1.04 (15)		1.47 (13)		0.98 (18)	
	Bacillus subtilis			1.68 (5)			
Yersinia ruckeri (1)	No reliable ID	1.67 (2)				1.68 (2)	
	Yersinia ruckeri			1.88 (9)			
	Yersinia pseudotuberculosis	1.75 (8)		1.82 (6)			
	Yersinia pestis					1.82 (16)	
	Yersinia enterocolitica	1.72 (2)		1.88 (3)			
Yersinia pseudotuberculosis (3)	No reliable ID	1.64 (1)					
	Yersinia pseudotuberculosis	2.04 (38)	76.9 (30)	2.15 (53)	86.8 (46)		
	Yersinia pestis					1.96 (53)	35.8 (19)
Yersinia enterocolitica (2)	No reliable ID					1.64 (7)	
	Yersinia enterocolitica	2.18 (17)	58.3 (14)	2.3 (36)	100 (36)		
	Yersinia pseudotuberculosis	2.14 (7)	29.1 (7)				
	Yersinia pestis					1.97 (29)	33.3 (12)
Francisella philomiragia (3)	No reliable ID	1.14 (39)		1.43 (14)		1.16 (54)	
	Francisella philomiragia			1.94 (40)	22.2 (12)		
Francisella novicida (2)	No reliable ID	1.1 (24)		1.36 (36)		1.45 (29)	
	Francisella tularensis					1.73 (6)	
Haemophilus influenzae (1)	No reliable ID	1.56 (5)				1.04 (18)	
	Haemophilus influenzae	2.1 (10)	60 (9)	2.19 (18)	83.3 (15)		
Burkholderia thailandensis (1)	No reliable ID	1.36 (15)					
	Burkholderia thailandensis			2.11 (18)	94.4 (17)		
	Burkholderia pseudomallei					1.96 (11)	22.2 (4)
	Burkholderia mallei					1.99 (7)	16.6 (3)
Burkholderia cepacia (1)	No reliable ID					1.34 (18)	
	Burkholderia cepacia complex	2.05 (15)	53.3 (8)				
	Burkholderia cepacia			2.17 (12)	66.6 (12)		
	Burkholderia cenocepacia			2.01 (2)	5.6 (1)		
	Burkholderia pyrrocinia			2.07 (4)	11.1 (2)		
Burkholderia cenocepacia (1)	No reliable ID					1.27 (18)	
	Burkholderia cepacia complex	2.04 (15)	60 (9)				
	Burkholderia cenocepacia			2.24 (18)	100 (18)		
Burkholderia multivorans (1)	No reliable ID					1.42 (18)	
	Burkholderia multivorans	2.18 (15)	73.3 (11)	2.14 (18)	77.7 (14)		
Stenotrophomonas maltophilia (1)	No reliable ID					0.95 (18)	
	Stenotrophomonas maltophilia	2.02 (15)	53.3 (8)	2.22 (18)	94.4 (17)		
Clostridium perfringens (1)	No reliable ID	1.0 (3)				1.05 (18)	
	Clostridium perfringens	2.22 (12)	80 (12)	2.31 (18)	100 (18)		
Clostridium difficile (1)	No reliable ID	0.97 (3)				1.04 (17)	
	Clostridium difficile	2.14 (12)	46.7 (7)	2.19 (17)	100 (17)		
Clostridium septicum (1)	No reliable ID	1.12 (15)				1.06 (18)	
	Clostridium septicum			2.30 (18)	100 (18)		
Clostridium sordellii (1)	No reliable ID	1.07 (15)				1.09 (18)	
	Clostridium sordellii			2.12 (18)	83.3 (15)		
Clostridium innocuum (1)	No reliable ID	1.18 (15)		1.55 (2)		1.06 (18)	
	Clostridium innocuum			2.20 (16)	83.3 (15)		
Clostridium butyricum (1)	No reliable ID	1.10 (15)		1.36 (3)		0.98 (18)	
	Clostridium butyricum			2.32 (15)	77.8 (14)		
Brucella neotomae (1)	No reliable ID	1.16 (15)		1.3 (18)			
	Brucella melitensis					1.96 (18)	22.2 (4)
Brucella ovis (1)	No reliable ID	1.06 (15)		1.32 (18)		1.36 (1)	
	Brucella melitensis					1.88 (17)	11.1 (2)
Ochrobactrum anthropi (1)	No reliable ID	1.04 (15)		1.69 (1)		1.15 (18)	
	Ochrobactrum spp.			1.96 (13)			
	Orchrobactrum intermedium			1.82 (1)			
Brucella pinnipedialis (1)	No reliable ID	1.13 (15)		1.43 (15)		1.63 (3)	
	Brucella melitensis					1.81 (15)	
	Ochrobactrum tritici			1.77 (3)			
Brucella ceti (1)	No reliable ID	1.1 (13)		1.34 (18)			
	Brucella melitensis					1.98 (18)	38.9 (7)
Oligella ureolytica (1)	No reliable ID	1.27 (2)		1.66 (1)		1.05 (18)	
	Oligella ureolytica	1.88 (13)	20 (3)	1.86 (17)	22.2 (4)		

**TABLE 5:** Identification of near neighbors by Vitek MS

Organism (no. of strains tested)	Reported identification	IVD library		RUO library	
		Mean score(s) (no. of replicates)	Species-level ID% (≥60%) (no. of replicates)	Mean score (no. of replicates)	Species-level ID % (≥60%) (no. of replicates)
Bacillus thuringiensis (1)	No identification			0 (1)	
	Bacillus mycoides/Bacillus cereus/Bacillus thuringiensis	33.3/33.3/33.3 (9)			
	Bacillus cereus group			81.6 (8)	
Bacillus circulans (1)	No identification	0 (7)		0 (7)	
	Bacillus circulans			45 (2)	
	Serratia rubidaea/Mycobacterium smegmatis	25/25 (1)			
	Escherichia coli	99.9 (1)	11.1 (1)		
Bacillus cereus (1)	No identification	0 (3)		0 (3)	
	Bacillus mycoides/Bacillus cereus/Bacillus thuringiensis	33.3/33.3/33.3 (6)			
	Bacillus cereus group			95.3 (5)	
	Capnocytophaga ochacea/C. sputigena			78.4 (1)	
Bacillus mycoides (1)	No identification	0 (3)		0 (1)	
	Bacillus mycoides/Bacillus cereus/Bacillus thuringiensis	33.3/33.3/33.3 (6)			
	Bacillus cereus group			91.5 (3)	
	Bacillus weihenstephanensis			84.7 (5)	55.6 (5)
Bacillus megaterium (1)	No identification			0 (2)	
	Bacillus megaterium	94.8 (9)	100 (9)	84.2 (3)	33.3 (3)
	Bacillus megaterium/Bacillus coagulans/Bacillus amyloliquefaciens			82.4 (3)	
	Bacillus coagulans/Bacillus megaterium			76.6 (1)	
Bacillus subtilis (1)	No identification			0 (6)	
	Bacillus amyloliquefaciens/Bacillus subtilis	50/50 (9)			
	Bacillus subtilis			82 (3)	33.3 (3)
Yersinia ruckeri (1)	No identification	0 (5)			
	Yersinia ruckeri	99.7 (4)	44.4 (4)		
	Yersinia spp.			85.7 (9)	
Yersinia pseudotuberculosis (3)	No identification	0 (1)			
	Yersinia pseudotuberculosis	99.6 (20)	95.2 (20)	91.9 (20)	95.2 (20)
	Yersinia enterocolitica			94.1 (1)	4.8 (1)
Yersinia enterocolitica (2)	No identification	0 (1)			
	Yersinia enterocolitica	99.9 (8)	66.7 (8)	97.5 (11)	91.7 (11)
	Yersinia pseudotuberculosis			86 (1)	
	Yersinia pseudotuberculosis/Yersinia enterocolitica	49.1/50.9 (3)			
Francisella philomiragia (3)	No identification	0 (27)		0 (25)	
	Microsporum canis			76.5 (1)	3.7 (1)
	Enterococcus spp.			81.2 (1)	3.7 (1)
Francisella novicida (2)	No identification	0 (16)		0 (8)	
	Francisella tularensis			92.2 (10)	55.6 (1)
	Erwinia rhapontici	75.1 (1)	5.6 (1)		
	Vibrio alginolyticus	81.2 (1)	5.6 (1)		
Haemophilus influenzae (1)	No identification	0 (4)		0 (6)	
	Haemophilus influenzae	99.9 (4)	44.4 (4)	93.5 (3)	33.3 (3)
	Haemophilus influenzae/Haemophilus haemolyticus/Streptococcus mitis/Streptococcus oralis	33/33/33 (1)			
Burkholderia thailandensis (1)	No identification	0 (9)		0 (5)	
	Burkholderia spp.			75.9 (3)	
	Staphylococcus epidermidis			75 (1)	
Burkholderia cepacia (1)	No identification	0 (4)		0 (1)	
	Burkholderia cepacia	99.4 (2)	28.6 (2)	83 (1)	11.1 (1)
	Burkholderia spp.			85 (7)	
	Burkholderia cepacia/Bacillus vietnamensis	50/50 (1)			
Burkholderia cenocepacia (1)	No identification	0 (3)		0 (5)	
	Burkholderia cepacia	99.8 (5)	55.6 (5)		
	Burkholderia spp.			88.2 (4)	
	Burkholderia cepacia/Bacillus vietnamensis	50.0/49.9 (1)			
Burkholderia multivorans (1)	No identification	0 (3)		0 (4)	
	Burkholderia multivorans	88.8 (6)	55.6 (5)	87.6 (3)	33.3 (3)
	Burkholderia spp.			87.1 (2)	
Stenotrophomonas maltophilia (1)	No identification	0 (2)		0 (3)	
	Stenotrophomonas maltophilia	99.9 (7)	77.8 (7)	93 (6)	66.7 (6)
Clostridium perfringens (1)	Clostridium perfringens	99.9 (9)	100 (9)	99.3 (9)	100 (9)
Clostridium difficile (1)	No identification	0 (3)		0 (1)	
	Clostridium difficile	96.5 (6)	66.7 (6)	97.4 (8)	88.9 (8)
Clostridium septicum (1)	No identification			0 (1)	
	Clostridium septicum	99.9 (9)	100 (9)	86.2 (8)	88.9 (8)
Clostridium sordellii (1)	No identification			0 (9)	
	Clostridium sordellii	92.6 (8)	88.9 (8)		
	Clostridium sordellii/Listeria monocytogenes	58.6/41.4 (1)			
Clostridium innocuum (1)	No identification	0 (8)		0 (7)	
	Citrobacter freundii			78.1 (1)	11.1 (1)
	Candida norvegensis			78 (1)	11.1 (1)
	Staphylococcus aureus	94.8 (1)	11.1 (1)		
Clostridium butyricum (1)	Clostridium butyricum	99.9 (6)	100 (6)	99.1 (9)	100 (9)
Brucella neotomae (1)	No identification	0 (9)			
	Brucella spp.			89.6 (9)	
Brucella ovis (1)	No identification	0 (9)		0 (3)	
	Brucella spp.			84 (6)	
Ochrobactrum anthropi (1)	No identification	0 (5)		0 (1)	
	Ochrobactrum anthropi	99.9 (4)	44.4 (4)	85.3 (3)	33.3 (3)
	Ochrobactrum spp.			88.8 (5)	
Brucella pinnipedialis (1)	No identification	0 (5)			
	Brucella spp.			89.6 (6)	
	Enterobacter asburiae/Ochrobactrum anthropi/Enterobacter cloacae/Vibrio parahaemolyticus	25/25/25/25 (1)			
	Ochrobactrum anthropi	99.9 (3)	33.3 (3)	81.6 (1)	11.1 (1)
	Ochrobactrum spp.			81 (2)	
Brucella ceti (1)	No identification	0 (9)		0 (1)	
	Brucella spp.			87.8 (7)	
Oligella ureolytica (1)	No identification	0 (3)		0 (4)	
	Oligella ureolytica	99.9 (5)	55.6 (5)	81.7 (3)	33.3 (3)
	Oligella spp.			78.9 (2)	
	Listeria seeligeri/Oligella ureolytica	43.2/56.7 (1)			

The Bruker IVD and RUO software did not correctly identify any of the BT agents. This is to be expected since BT agents are not included in the software. However, the IVD and RUO libraries incorrectly identified 11.9% and 16.2% of the isolates, respectively. The IVD software misidentified 73.8% of the Yersinia pestis extracts as Y. pseudotuberculosis, and the RUO software misidentified 8.3% of the Bacillus anthracis extracts, 81.5% of the Y. pestis extracts, 9.3% of the Burkholderia mallei extracts, and 5.6% of the B. pseudomallei extracts. Some participants also reported unvalidated identifications of B. cereus for the B. anthracis extracts and B. thailandensis for B. pseudomallei or B. mallei using the IVD software. The Bruker SR library correctly identified 52.5% of the BT extracts tested; 9.6% of the results were incorrect identifications, and the remaining 38.1% gave no reliable identification. Some extracts of B. pseudomallei were identified as B. mallei and vice versa. A total of 56 of 107 (52.3%) Brucella spp. were misidentified as B. melitensis; however, B. melitensis was the only species represented in the library.

Among the near-neighbor isolates, the Bruker IVD software misidentified 1.4% of the extracts, with all 7 errors identifying Y. enterocolitica as Y. pseudotuberculosis. The RUO software misidentified 1.1% of the extracts, with over half of the errors accounted for by B. thuringiensis being identified as B. cereus. The SR software misidentified 10.7% of the extracts. B. thuringiensis (38.9%) and B. cereus (50%) were misidentified as B. anthracis; Y. pseudotuberculosis (35.8%) and Y. enterocolitica (33.3%) were misidentified as Y. pestis; B. thailandensis (38.9%) was identified as either B. mallei or B. pseudomallei; and 12% of near neighbors of Brucella were identified as B. melitensis.

The Vitek IVD library did not correctly identify any of the BT agents but incorrectly identified 16.2% of the isolates. While several of the BT agents are in the RUO library, only 3.3% of extracts were correctly identified; F. tularensis was the only BT agent identified, with 11 of 45 (24.4%) extracts identified correctly. The RUO library incorrectly identified 7.5% of the extracts. Y. pestis was the most frequently misidentified organism, with 60.7% and 33.3% extracts being identified as Y. pseudotuberculosis by the IVD and RUO software, respectively. While the RUO software did not identify any of the Brucella extracts to the species level, it did correctly identify them to the genus level 56.9% of the time.

The IVD and RUO libraries misidentified 2.3% and 7% of the near-neighbor extracts, respectively. The RUO library incorrectly identified 55.6% of Francisella novicida extracts as F. tularensis.

## DISCUSSION

MALDI-TOF MS presents clinical laboratories with a new tool that has the potential to rapidly and accurately identify organisms in a cost-effective manner; however, this technology also presents new challenges. Highly pathogenic organisms may present hazards to the laboratory staff during the preparation and testing of samples. Validation of identification systems also poses a challenge in that access to many highly pathogenic organisms is regulated by the Select Agent Program and, thus, these agents are not available to clinical laboratories to assess the limitations of the software libraries.

Use of MALDI-TOF MS for the rapid identification of naturally or intentionally released risk group 3 organisms in a biosafety level 2 (BSL2) environment makes inactivation a critical step to limit exposure risk for laboratorians. In addition to the sample preparation methods described by instrument manufacturers, several other methods have been proposed to inactivate highly pathogenic organisms, including the use of trifluoroacetic acid (TFA), ethanol, gamma irradiation, centrifugation, and filtration. Nonetheless, there are disadvantages associated with these methods. Treatment with 80% TFA for 30 min, for instance, has been shown to inactivate vegetative cells but failed to consistently kill spores of B. cereus and B. subtilis (5). The addition of centrifugation and filtration through a 0.22-μm-pore-size membrane removed all remaining viable organisms and spores. However, the final preparation required a 1:10 dilution in water, which may decrease analytical sensitivity, and the high toxicity of TFA may also preclude its use in clinical laboratories. Gamma irradiation has been shown to successfully inactivate organisms (10, 11), but decreased peak intensities led to lower identification scores, and the availability of a γ source in clinical laboratories makes this approach untenable. Exposure to 70% ethanol for 5 min has been shown to inactivate non-spore-forming near-neighbor organisms but failed to inactivate B. cereus and C. sporogenes (4). TFA extraction and a tube extraction method utilizing ethanol-formic acid-acetonitrile rendered 14 of 15 bacterial strains nonviable; B. anthracis A100 survived, but all extracts were nonviable following the addition of centrifugal filtration through a 0.1-μm-pore-size filter (6). Tracz et al. (7) showed that 3 of 31 Bacillus spp., including one B. anthracis strain and two B. thuringiensis strains, survived tube extraction, but the extracts were rendered nonviable following the addition of a filtration step.

This study showed that some of the BT agents survived the direct and on-plate formic acid sample preparation techniques widely used by clinical laboratories. These results differ from findings by Cunningham and Patel (4), who reported that all isolates tested were nonviable following treatment with 70% formic acid (on-plate sample preparation). However, the studies differed in the isolates tested. Vitek's on-plate formic acid sample preparation method utilizes 25% formic acid, whereas the present study used 70% formic acid as recommended by Bruker; thus, the results of organism inactivation using 70% formic acid may differ from those obtained using 25% formic acid. The operator's technique could also influence organism viability if the spotted organism is not completely covered by formic acid or the spot is not entirely encased by matrix. While none of the isolates tested in this study survived the tube extraction method, other investigators (6, 7) have shown that some isolates of B. anthracis and B. thuringiensis may survive the tube extraction procedure; those investigators recommended the use of a filtration step for added safety. The results of this and previous studies indicate that several inactivation procedures may be successful; however, intraspecies differences may make one strain more resistant to inactivation than others. The addition of a filtration step combined with the manufacturer's tube extraction procedure provides an increased margin of safety to ensure that samples contain no viable organisms. On the basis of this information, the American Society for Microbiology document “Sentinel Level Clinical Laboratory Protocols for Suspected Biological Threat Agents and Emerging Infectious Diseases” (www.asm.org/index.php/science-skills-in-the-lab/sentinel-guidelines) recommends that laboratories using MALDI-TOF MS for identification of suspect BT agents should use the tube extraction method followed by filtration through a ≤0.2-μm-pore-size filter for suspected BT agents. Filtration of DNA preparations of B. anthracis spores for PCR through the use of a 0.1-μm-pore-size filter prior to testing has been shown to render samples safe for testing outside BSL3 containment (12); this practice is widely used by state public health laboratories participating in the Laboratory Response Network (LRN) and should be extended to extracts of suspected highly pathogenic organisms prepared for MALDI-TOF MS.

Accurate assays for the identification of highly pathogenic organisms are critical for timely treatment, for decreasing laboratory exposures, and for instituting appropriate public health interventions that may be associated with an intentional release. In the United States, naturally occurring cases of brucellosis (115 in 2010), tularemia (314 in 2015), and plague (16 in 2015) reported to CDC pose additional hazards and diagnostic challenges for clinical laboratories. A European interlaboratory ring trial testing the ability of MALDI-TOF MS to identify six BT agents and four near neighbors showed an average accuracy of 77% (11). However, in 5 of the 12 participating laboratories that utilized Bruker software alone, the accuracies were 46.7% for six BT agents and 50% for the near neighbors. For the single Vitek participant, the accuracies were 66.7% for the BT agents and 100% for near neighbors. Another study (7) that looked at 57 isolates representing nine potential BT organisms showed an accuracy of 61.4% using the Bruker RUO and SR libraries. Those studies are in general agreement with the findings of the present study. In addition, both of those studies showed that the combination of the manufacturers' libraries and in-house libraries improved accuracy to >93% (11) and 100% (7). The results of those studies indicate the need for additional spectra in the commercial databases to improve identification accuracy.

Accurate results employing mass spectrometry require good sample preparation and a well-developed database. Several studies have looked at improving accuracy by optimizing specimen preparation and altering the manufacturer's criteria for genus- and species-level identification. Studies have suggested scores of ≥1.7 for Gram-positive organisms (13), ≥1.9 for enteric Gram-negative bacilli (14), ≥1.8 (15), and ≥1.9 (16) for anaerobic bacteria and even species-specific cutoff scores (17) to improve identification accuracy. The accuracy of identification reported in the present study might also increase if cutoff scores were optimized. The mean score for many the BT agents was near the cutoff value of ≥2.0, and a decrease to even ≥1.9 would have significantly improved identification to the species level. Identification accuracy can be improved by using phenotypic characteristics combined with MALDI-TOF results to make a final identification. CLSI recommends the use of Gram stain characteristics, colony morphology, rate of growth, culture conditions, and biochemical and/or antimicrobial susceptibility test (AST) results (9). For example, in this study, a Gram stain performed for the Vitek extracts would have detected 19% of the IVD misidentifications and 36.4% of the RUO misidentifications.

The sample preparation method may also have affected the accuracy of the study results. While our limited data suggest that the ethanol/formic acid extraction method employed here is compatible with the Vitek MS system, further studies to validate this extraction method are warranted. The interlaboratory effects of sample preparation technique were minimized in this study since all of the extracts were prepared in a total of four laboratories; however, storage and handling of the extracts could affect spectral quality. Our study showed no effect on identification scores for up to 45 days when extracts were stored at −20°C; however, some of the study participants analyzed extracts well beyond 45 days of storage. This may have affected spectral quality for some extracts, decreasing specimen scores and resulting in lower accuracy. However, it should also be noted that extracts for C. perfringens, C. septicum, C. sordellii, B. cepacia, and Y. enterocolitica were correctly identified by all Bruker participants and that B. megaterium, C. perfringens, and C. septicum were correctly identified by all Vitek participants regardless of the time between sample preparation and analysis. When the data for the identification of BT agents by the SR library were reanalyzed based on test date, we found that 13.8% (96/697) of the extracts were tested beyond 45 days. Inclusion of only those extracts tested within 45 days increased the overall accuracy from 52.5% to 55.2%. While the identification accuracy for most agents increased, the accuracy for Clostridium botulinum and B. mallei decreased slightly. This suggests that testing beyond 45 days resulted in decreased spectral quality for some extracts whereas others were left unaffected. Additional studies conducted at a single laboratory are necessary to determine how storage time/temperature and genus/species affect spectral stability. These studies may have a significant impact on future multilaboratory studies and proficiency testing using prepared extracts.

The Bruker and Vitek IVD databases both exclude BT agents; Vitek covers some of the agents in the RUO database, and Bruker requires purchase of a separate database to identify these agents. While the Vitek RUO database failed to identify most of the agents to the species level, it provided genus-level (e.g., Brucella, Burkholderia), group-level (B. cereus group), or split-organism (B. thuringiensis/B. cereus/B. mycoides) identifications for some of the organisms. This level of identification may decrease exposure risks in clinical laboratories if they recognize software limitations and use appropriate supplemental testing procedures such as those outlined in the American Society for Microbiology (ASM) document “Sentinel Level Clinical Laboratory Protocols for Suspected Biological Threat Agents and Emerging Infectious Diseases.” For example, 51 health care workers were exposed to B. melitensis in two incidents within 2 months in New York City (18), in part because both laboratories attempted identification using MALDI-TOF MS and the genus Brucella was not part of the instrument's database. Manufacturers should consider inclusion of the BT agents in their IVD/RUO databases for identification to the genus level or the species level or both together and specific instructions that results should be confirmed by other methods. In this study, the most frequently misidentified organism was Y. pestis. Differentiation from Y. pseudotuberculosis is problematic because Y. pestis evolved from Y. pseudotuberculosis only recently (19). Until that differentiation is possible, manufacturers may want to consider a disclaimer for the identification of both organisms. Until databases are updated, laboratories should clearly note limitations in their procedures and may want to consider the use of well-curated external databases like CDC's MicrobeNet. Currently the Bruker RUO library offers a “matching hints” disclaimer, which in some instances may assist a user in electing to follow the ASM recommended guidelines. However, the “matching hints” disclaimers also indicate the use of repeat testing with fresh material for Bacillus spp., which may increase exposure risk.

Implementation of MALDI-TOF MS in clinical laboratories poses some significant issues that should be addressed in a risk assessment and with validation studies. Laboratories should consider the hazards that preparing and testing potential BT agents and other agents easily transmitted by aerosol pose for health care workers. Since BT agents are not readily available for validation studies, laboratories should also be aware of software limitations and common misidentifications. Partial identifications or misidentifications resulting from the use of IVD (including unclaimed identifications) and RUO software in this study include B. anthracis identified as B. cereus, B. cereus group, or B. thuringiensis/B. cereus/B. mycoides; Y. pestis identified as Y. pseudotuberculosis; B. mallei or pseudomallei identified as B. thailandensis or B. multivorans; and C. botulinum identified as C. sporogenes. Until the software libraries are capable of reliable identification of the BT agents, clinical laboratories should continue to rely on basic phenotypic characteristics like colony morphology, growth rate, spot tests, and Gram stain to determine which identification algorithm is appropriate. When phenotypic characteristics indicate a potential BT agent, clinical laboratories should utilize the ASM Sentinel Level Clinical Laboratory Protocols prior to attempting identification with MALDI-TOF MS.

## MATERIALS AND METHODS

### Safety study.

Isolates of Bacillus anthracis Sterne, Brucella abortus strain 19, Burkholderia thailandensis ATCC 70038, Clostridium botulinum (clinical isolates of toxin types A, B, and E), Clostridium perfringens WAL-14572, Francisella tularensis subspecies holarctica LVS, and Yersinia pestis A1122 were prepared for testing using the direct colony, on-plate formic acid extraction, and ethanol/formic acid tube extraction methods according to Bruker's user's manual (20) with the following modifications: (i) to obtain uniform spotting, samples for the direct colony and on-plate extraction methods were prepared in high-performance liquid chromatography (HPLC)-grade water with turbidity equivalent to a 1 to 2 McFarland standard; (ii) samples for the tube extraction were prepared in HPLC-grade water with turbidity equivalent to a 3 to 4 McFarland standard; and (iii) 1-μl aliquots were spotted onto sterile 15-mm-diameter no. 1 glass coverslips instead of the MALDI target.

A total of nine coverslips, representing a MALDI target, were prepared for each organism, and three were used for each extraction method at five participating laboratories. The coverslips were allowed to air dry. One coverslip was placed into 10 ml of brain heart infusion (BHI) broth supplemented or conditioned as needed to support organism growth. This coverslip (referred to as the “Spot” coverslip) served as a control to determine the effects of drying and air exposure (for anaerobes) on viability. A second coverslip was placed into a tube of BHI broth that contained all the reagents used in the extraction (for example, 1 μl of 70% formic acid and 1 μl of α-cyano-4-hydroxycinnamic acid [HCCA] matrix for the on-plate extraction samples). This coverslip (referred to as the “Spot + Matrix” coverslip) was used to determine growth inhibition due to inadequate dilution of the extraction reagents in BHI broth. For the third coverslip, the extracted sample was overlaid with 1 μl of HCCA, allowed to air dry, and then placed into 10 ml BHI broth. This coverslip (referred to as the “Target” coverslip) represented a sample ready for MALDI analysis. The tubes were incubated using appropriate temperatures and conditions for 7 days (21 days for Brucella). Any tube showing turbidity was subcultured and the growth identified by Gram stain and morphology.

### Accuracy study.

Whenever possible, the strains utilized for the study were clinically relevant organisms selected from the inclusivity and exclusivity panels approved by the AOAC International Stakeholder Panel on Agent Detection Assays (SPADA) (21–23; AOAC International, unpublished data). No SPADA panels were developed for Brucella species or Clostridium botulinum, so strains of these species were selected based on availability and clinical relevance. BT agents used for the study are listed in Table S1 in the supplemental material along with their relationship to the SPADA panels and the presence of each genus and species in the software libraries tested.

Each isolate was prepared by performing Bruker's tube extraction in 10 replicates followed by filtering each extract through a 0.1-μm-pore-size centrifugal filter (Millipore Ultrafree—MC-VV Durapore polyvinylidene difluoride [PVDF]) for 2 min at 7,050 × *g*. The resulting extracts were pooled, mixed, divided into aliquots in 50-μl volumes, and stored at −20°C. Ten percent of the final pooled volume or 100 μl was tested to confirm sterility. Extracts were shipped on dry ice to the testing laboratories.

Participating laboratories were asked to test all extracts in triplicate on the same run using a freshly cleaned or disposable target within 45 days of extract preparation. A 1-μl volume of extract was applied to the target, allowed to dry, and then overlaid with 1 μl HCCA matrix. Spectra were generated using the run conditions programmed by the manufacturers. Six laboratories tested extracts on a Bruker MALDI Biotyper (Bruker Daltonics, Billerica, MA) equipped with one or more of the IVD (claim 1), RUO (claim 3; *n* = 5,687), and Security-Relevant (claim 1; *n* = 123) software libraries. Three laboratories tested extracts on the Vitek MS system (bioMérieux Inc., Durham, NC) equipped with the IVD (version 2.0) and RUO (version 4.12) software libraries.

Laboratories with IVD software were instructed to test extracts using each manufacturer's IVD protocol. Following completion of the run, the spectral data generated from the run were reanalyzed using all available software packages but performing the analysis with only one software package at a time. For laboratories with RUO software, spectra were generated in RUO mode. Each laboratory reported results using a spreadsheet listing the date tested, the software package used, the identification result, and the sample score. An identification result was considered accurate to the genus and species levels if the sample score was ≥2.0 for the Biotyper or the level of identification was ≥60% for the Vitek MS.

## Supplementary Material

Supplemental material
