# Supplementary material for: Safety and Accuracy of Matrix-Assisted Laser Desorption Ionization–Time of Flight Mass Spectrometry for Identification of Highly Pathogenic Organisms
Source: J Clin Microbiol. 2017 Nov 27;55(12):3513–29. doi: 10.1128/JCM.01023-17 (PMC5703816; doi:10.1128/JCM.01023-17)
Supplement: Supplemental material [file supp_55_12_3513__index.html]

Supplemental material 

# Safety and Accuracy of Matrix-Assisted Laser Desorption Ionization–Time of Flight Mass Spectrometry for Identification of Highly Pathogenic Organisms

## Supplemental material

- Supplemental file 1 -

  Table S1 (Near-neighbor isolates, their relationship to SPADA panels, and inclusion in software databases to the genus and species levels)

  PDF, 49K
