# Supplementary material for: Safety and Accuracy of Matrix-Assisted Laser Desorption Ionization–Time of Flight Mass Spectrometry for Identification of Highly Pathogenic Organisms
Source: J Clin Microbiol. 2017 Nov 27;55(12):3513–29. doi: 10.1128/JCM.01023-17 (PMC5703816; doi:10.1128/JCM.01023-17)
Supplement: Supplemental material [file JCM.01023-17_zjm012175730s1.pdf]

Table SI-1 Near neighbor isolates, their relationship to SPADA panels and inclusion in software databases to the genus and species level.

Species	Strain	SPADA Panel	Bruker IVD	Bruker IVD Unclaimed	Bruker RUO	Bruker SR	Vitek IVD	Vitek IVD Unclaimed	Vitek RUO
Bacillus cereus	ATCC 5416	x		x	x			g	x
Bacillus mycoides	ATCC 6462	x			x			g	x
Bacillus megaterium	ATCC 14581	x		x	x			x	x
Bacillus subtilis	ATCC 6051T	x			x			g	x
Bacillus thuringiensis	ATCC 33679			x	x			g	x
Bacillus circulans	ATCC 5416			x	x				x
Francisella philomiragia	ATCC 25015	x		x	x				x
Francisella philomiragia	ATCC 25016	x		x	x				x
Francisella philomiragia	ATCC 25017	x		x	x				x
Francisella novicida	NR-574, U112	x							
Francisella novicida	NR-575								
Haemophilus influenzae	ATCC 10211		x		x		x		x
Yersinia ruckeri	YERS063	x		x	x			x	x
Yersinia pseudotuberculosis	YPIII	x	x		x		x		x
Yersinia pseudotuberculosis	Pa3606	x	x		x		x		x
Yersinia pseudotuberculosis	IB	x	x		x		x		x
Yersinia enterocolitica	WA	x	x		x		x		x
Yersinia enterocolitica	2516-87	x	x		x		x		x

Clostridium perfringens	ATCC 13124		x		x		x		x
Clostridium difficile	ATCC 9689		x		x		x		x
Clostridium septicum	ATCC 9714			x	x			x	x
Clostridium sordellii	ATCC 9715			x	x			x	x
Clostridium innocuum	ATCC 14501			x	x				y
Clostridium butyricum	ATCC 19398			x	x			x	x
Brucella neotomae	ATCC 23459								x
Brucella ovis	NVSL								x
Brucella pinnipedialis	NVSL								y
Brucella ceti	NVSL								y
Oligella ureolytica	ATCC 43535		x		x		x		x
Ochrobactrum anthropi	ATCC 49188			x	x		x		x
Burkholderia thailandensis	ATCC 700388	x		x	x				x
Burkholderia cepacia	ATCC 25416		x		x			x	x
Burkholderia cenocepacia	ATCC BAA-245		x		x		x		x
Burkholderia multivorans	BAA-247		x		x				x
Stenotrophomonas maltophilia	ATCC 13637		x		x		x		x

x: included in panel or software library; y: included in software library to genus level only; g: reported as an organism group
